# Hear, Hear for Notch: Control of Cell Fates in the Inner Ear by Notch Signaling

**DOI:** 10.3390/biom10030370

**Published:** 2020-02-28

**Authors:** Rogers Brown, Andrew K. Groves

**Affiliations:** 1 Program in Developmental Biology; Baylor College of Medicine, Houston, TX 77030, USA; rmabrown2@gmail.com; 2 Department of Neuroscience; Baylor College of Medicine, Houston, TX 77030, USA; 3 Department of Molecular and Human Genetics, Baylor College of Medicine, Houston, TX 77030, USA

**Keywords:** inner ear, Notch signaling, cochlea, hair cells, supporting cells, cochleovestibular ganglion, lateral inhibition

## Abstract

The vertebrate inner ear is responsible for detecting sound, gravity, and head motion. These mechanical forces are detected by mechanosensitive hair cells, arranged in a series of sensory patches in the vestibular and cochlear regions of the ear. Hair cells form synapses with neurons of the VIIIth cranial ganglion, which convey sound and balance information to the brain. They are surrounded by supporting cells, which nourish and protect the hair cells, and which can serve as a source of stem cells to regenerate hair cells after damage in non-mammalian vertebrates. The Notch signaling pathway plays many roles in the development of the inner ear, from the earliest formation of future inner ear ectoderm on the side of the embryonic head, to regulating the production of supporting cells, hair cells, and the neurons that innervate them. Notch signaling is re-deployed in non-mammalian vertebrates during hair cell regeneration, and attempts have been made to manipulate the Notch pathway to promote hair cell regeneration in mammals. In this review, we summarize the different modes of Notch signaling in inner ear development and regeneration, and describe how they interact with other signaling pathways to orchestrate the fine-grained cellular patterns of the ear.

## 1. Introduction

The vertebrate inner ear is responsible for detecting sound and linear and angular acceleration and conveying these signals to the brain. It does so with mechanosensitive hair cells, capable of responding to atomic level displacements. These secondary receptor cells form synapses with neurons of the VIIIth (cochleovestibular) ganglion, which are also generated by the inner ear. Hair cells and neurons arise within a complex three-dimensional structure and are highly patterned on a cellular level [1,2,3]. This elaborate sensory organ arises from a thickening of the embryonic ectoderm termed the otic placode, which arises adjacent to the developing hindbrain shortly after the end of gastrulation. This thickened ectoderm rapidly invaginates to form a sphere of epithelial cells termed the otocyst, and it is from this simple sphere that the entire inner ear and its associated sensory hair cells and neurons is sculpted. The Notch signaling pathway is deployed at several points during the development of the ear, from the origin of the otic placode to the formation of mature mechanosensory sensory structures. The hair cells of the inner ear can be killed by loud noises, certain kinds of antibiotics and chemotherapeutic drugs, as well as the wear and tear of old age. Although mammals are unable to regenerate their hair cells—with the consequence that hearing loss is progressive and permanent—other vertebrate groups are capable of impressive feats of hair cell regeneration and functional recovery. The Notch pathway is redeployed during regeneration in these species and has led to the idea that manipulation of Notch signaling may be used to restore hair cells in mammals. Here, we review and analyze evidence for the different forms of Notch signaling during development of the inner ear. This subject has benefited greatly from the availability of knockouts and ear-specific conditional knockouts of many components of the Notch pathway, and a list of many of these mutants described throughout the text are collated in Table 1.

## 2. The First Steps in Ear Induction—How Notch Signals Regulate the Size of the Otic Placode

The otic placode that gives rise to the entire inner ear is one of a series of craniofacial placodes that form the olfactory epithelium, the entire inner ear, neurons in a variety of cranial sensory ganglia, and accessory sensory structures, such as the lens of the eye [4,5,6,7]. The development of this region, dubbed the pre-placodal region (PPR), is more fully reviewed elsewhere [7,8], but is characterized by expression of a common set of transcription factors (Six1, Eya2, and Foxi3). The PPR forms at the neural plate border region that gives rise to the neural tube, neural crest, placodes, and future cranial epidermis. At the end of gastrulation, the PPR receives a series of regionalized signals along its anterior–posterior axis that pattern it into individual placodes [9]. The otic placode forms from the PPR at the level of rhombomeres 4–6 of the hindbrain [10]. The earliest markers of the otic placode are the transcription factors Pax2 and Pax8 [10,11]. A large number of studies in different vertebrate species have concluded that members of the FGF signaling family are both necessary and sufficient to induce the otic placode from the PPR [4,12]. The particular members of the FGF family and the source of their production varies in different vertebrate classes—for example, FGF3 produced by the hindbrain and FGF10 expression in the cranial mesoderm cooperate to induce the otic placode in mammals [13]. Fate mapping studies of the Pax2-expressing lineage show that this region gives rise to all parts of the inner ear, as well as the epibranchial placodes and some epidermis [11].

Within the broad initial Pax2-expressing domain, further refinement is required to differentiate between the otic and epibranchial placodes. The strength and duration of FGF signaling play a role in determining otic placode fate, with proteins involved in attenuating FGF signaling, such as Sprouty2, Dusp6, and Dusp9, being rapidly upregulated in the otic placode [13,14,15]. At the same time as FGF signaling is attenuated, Wnt signals from the midline and neural plate direct Pax2-expressing cells towards an otic fate. Loss of Wnt signaling in this domain results in a significantly smaller otic placode, while driving constitutively active Wnt signaling throughout the Pax2 domain results in conversion of this entire domain to an otic fate [16]. Wnt signaling also controls the expression of the Notch ligand Jag1 within the otic placode, and this Notch signaling activity seems to be important for defining the otic placode and separating it from the Pax2-expressing ectoderm that will become epidermis and epibranchial placodes [17]. Disruption of Wnt signaling by conditional deletion of the canonical downstream transcription factor β-catenin results in a reduced expression pattern of the Notch ligand Jagged1 (Jag1), while constitutively active β-catenin results in the spread of Jag1 expression laterally from the neural plate throughout the Pax2-expressing domain [17]. Lef/Tcf binding sites that bind canonical Wnt signaling complexes are located upstream of the Jag1 promoter [18,19], suggesting that the induction of Jag1 by Wnt signaling may be direct. The expression of Jag1 in the otic placode is coincident with expression of the Notch1 receptor, likely through a positive-feedback mechanism known as Notch-mediated lateral induction. Notch1 expression expands laterally from the neural plate throughout the Pax2 domain in a similar pattern to Jag1 when β-catenin is constitutively activated, while a truncated otic cup with a smaller domain of Notch1 expression is seen after deletion of β-catenin in the Pax2 domain [17]. Gain- and loss-of-function experiments suggest that Jag1–Notch1 signaling in the otic placode enhances Wnt signaling during otic placode induction. Expression of the Notch1 receptor intracellular domain (N1ICD) throughout the Pax2-expressing domain expands otic markers and enhances Wnt signaling activity measured in Wnt reporter mice. Conversely, Notch1 mutants also have a smaller otic placode, similar to β-catenin conditional mutants [17]. Together, these observations lead to a model in which Notch signaling and Wnt signaling work together to refine the boundaries of the otic placode, with Notch signaling supplementing and strengthening Wnt activity in the parts of the otic placode furthest from the neural plate source of Wnt expression [20].

## 3. Notch Signaling Regulates Neurosensory Fate in the Inner Ear

**Figure 1:**
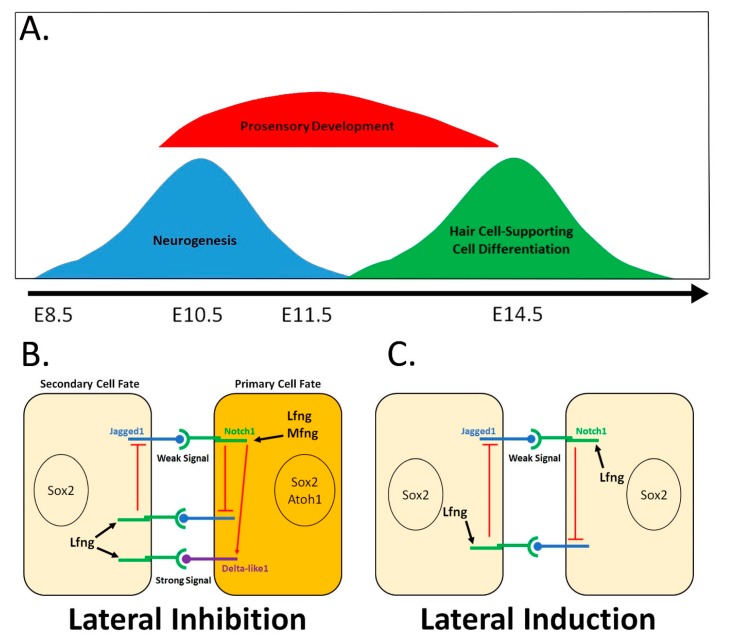
The inner ear uses two modes of Notch signaling during key phases in early inner ear development. (**A**) Once the otocyst has been formed, there are three phases during which Notch signaling plays an important role in determining cell fates in inner ear development: neurogenesis and hair cell differentiation, which utilize (**B**) the most familiar and more robust form of Notch signaling—lateral inhibition—and prosensory domain development, which employs (**C**) a lesser understood and weaker form of Notch signaling—lateral induction. (**B**) Lateral inhibition utilizes a negative feedback loop to differentiate between primary cell fates and secondary cell fates in the inner ear. (**C**) Lateral induction uses a positive feedback loop to promote a singular cell fate within a patch of cells in inner ear development.

As the otic placode begins to invaginate, the first sign of terminal differentiation is the formation and delamination of neuroblasts from the anterior and ventral region of the otic cup, termed the neurosensory domain [21]. This neurogenesis creates the VIIIth cranial ganglion (also known as the cochleovestibular ganglion), which later separates into its auditory and vestibular components, also known respectively as the spiral ganglion and Scarpa’s ganglion. The first signs of neurogenesis in the antero-ventral region of the otic cup is expression of the neurogenic transcription factor Neurog1, the Notch signaling ligand Dll1, and the Notch-modifying N-acetylglucosaminyltransferase, Lunatic Fringe (Lfng) [22,23,24,25]. As the otic cup completes its closure and invagination, this neurosensory domain generates neuroblasts, starting at embryonic day (E) 9.0 in mice and peaking a day later, with the bulk of neurogenesis being completed by E14.5 [22,23]. The neurogenic region is adjacent to a non-overlapping region of epithelium expressing Bmp4, Tbx1, and Lmx1 [22,25,26,27]. This region does not form neurons and instead generates sensory and non-sensory regions of the cochlea and vestibular canals. Tbx1 activity maintains this non-neurogenic region, and in the absence of Tbx1, this region forms a second ganglion complete with delaminating neurons. Otic neuroblasts delaminating from the neurosensory region divide once or twice, and then become post-mitotic [24]. Several days later, cells in this domain that do not delaminate to form the VIIIth ganglion contribute to the hair cells and supporting cells in the sensory maculae of the utricle and saccule in the vestibular system (Figure 1). This was demonstrated most directly by lineage tracing with Neurog1–CreER mice, which labels derivatives in the VIIIth ganglion and sensory epithelium of the utricle and saccule, but not other sensory organs in the inner ear [23,28]. This suggests that some cells that begin to express Neurog1 do not differentiate into neurons but are instead inhibited from this fate and later differentiate as sensory cells. Notch signaling has been shown to regulate both the neurogenic process and the development of the hair cells and supporting cells in the neurosensory domain of the inner ear [23,29].

The decision as to which cells will delaminate and segregate as neural precursors from the neurosensory domain is regulated by Notch-mediated lateral inhibition. In the neurosensory domain, slight and stochastic upregulation of the Neurog1 transcription factor in individual cells results in the expression of a Notch ligand, Dll1, in presumptive neuroblasts. These Dll1-expressing cells then signal to adjacent cells more strongly, and the activation of the Notch signaling pathway in neighboring cells results in their subsequent downregulation of Neurog1. Several lines of evidence support this lateral inhibition model. First, loss of Neurog1 results in the loss of Dll1 expression in the anterior otocyst [23]. Inhibition of Notch signaling in a chick otocyst with the gamma secretase inhibitor DAPT results in excessive delamination of neuroblasts from the otocyst, to the point where neuroblasts delaminate from the center of the otic vesicle as well as their normal anterior location [30]. The mind bomb (Mib) gene encodes a RING domain E3 ligase, which promotes Notch signaling through ubiquitylation and internalization of the Dll1 ligand [31]. Mind bomb alleles with inactivating point mutations in the RING domain cause an expansion of Dll1 expression and a doubling of the number of neuroblasts adjacent to the otocyst [32].

As described above, about 48 h after neurogenesis begins in the neurosensory domain, the domain begins to form hair cells. This is presaged by the appearance of two stripes of the hair cell transcription factor Atoh1, which expand over several days to form maculae of the utricle and saccule. As hair cells begin to differentiate, the Neurog1 domain contracts in complementary fashion [23]. Interestingly, in Atoh1 mutant mice, which cannot produce hair cells, the Neurog1 domain does not contract, but instead generates neurons over large regions of the utricle and saccule that would normally produce hair cells [23]. Conversely, in Neurog1 mutant mice, expansion of the Atoh1 domain is accelerated, leading to increases in hair cell formation into regions that would normally generate neurons [23]. These results are consistent with a phenomenon reported in the nervous system, where cross-inhibition between bHLH factors controls commitment of progenitor cells to alternative fates in various brain regions [33,34]. Since both Atoh1 and Neurog1 are transcription factors, the cross-repression of these two genes must occur through cell surface or secreted intermediaries. Since Neurog1-expressing neuroblasts also express Dll1 [29], and nascent hair cells express both Dll1 and Jag2, it is possible that these two distinct populations use Notch signaling to repress transcription factors (Atoh1 or Neurog1), promoting the alternative fate (hair cells or neuroblasts) in the neurosensory domain, although this has yet to be formally tested.

## 4. Notch-Mediated Lateral Induction Plays A Key Maintenance Role in Prosensory Domain Development

As the otic cup closes to form the otocyst and begins to generate the neuroblasts of the cochleo-vestibular ganglion, it also begins to establish regions of epithelium that will ultimately give rise to the sensory organs of the inner ear containing mechanosensitive hair cells [21]. These regions, known as prosensory domains, are associated with a thickening of the otocyst walls due to widening of the medio-lateral length of otic epithelial cells, and the expression of a key set of genes integral to proper sensory organ development. Mammals contain six prosensory domains that will generate the mature sensory organs of the ear: three cristae at the base of each semicircular canal that detect angular acceleration of the head, two maculae (the utricular and saccular maculae) that detect linear acceleration due to motion and gravity, and the organ of Corti running the length of the cochlear duct that detects sound. The locations of the six prosensory regions are specified by a number of signaling pathways, including BMP, FGF, Shh, Wnt, and retinoic acid [20]. These signals help specify the body plan of the embryo, and are used to set the cardinal axes of the otocyst in a similar way—for example, Shh signaling from the floor plate and notochord specifies the dorso-ventral axis of the nervous system, but also impart a dorso-ventral character on the otocyst [35,36]. Similarly, a gradient of retinoic acid, which imparts an anterior–posterior identity on the nervous system and paraxial mesoderm, also patterns the otocyst in the A–P axis. These signals all help to establish a three-dimensional grid of positional information, which the otocyst uses to generate prosensory domains in the correct location. The specific signals and mechanisms of action are further reviewed elsewhere [20,37]; here we shall focus on the role of Notch signaling in prosensory development.

All prosensory domains of the otocyst express the SRY-box transcription factor Sox2 and the Notch ligand Jag1. The LIM homeobox transcription factor Lmx1a is expressed in the non-sensory regions of the otocyst, and mutation of this gene causes a failure of many of the prosensory patches to correctly separate and individuate [27,38,39]. The vestibular prosensory domains can then be further distinguished by the expression of genes specifically expressed in the future cristae and maculae. The cristae express BMP4 and less exclusively FGF10 (which is also weakly expressed in the utricle by E11.5), which both seem to play a major role in guiding semi-circular canal development [2,25,40,41,42]. BMP4 is expressed in two broad vestibular anterior and posterior patches at E10.5, which gradually refine, with the anterior patch elongating and splitting to forms the anterior and lateral cristae prosensory domains by E11.5 [42]. The maculae are largely identified by their exclusive vestibular expression of Lfng [25], which as mentioned above is expressed in an anterio-ventral portion of the otocyst at E10.5, eventually restricts by E11.5, and is expressed in the primitive utricle and saccule by E12.5 (Figure 1).

An interesting feature of the canonical prosensory markers, Sox2 and Jag1, and the canonical non-sensory marker Lmx1a, is that they are all expressed in a broad pattern throughout the otic cup as it closes [17,26,43]. However, as prosensory development begins, the expression of all three genes shifts and eventually separates over a 48 h period until Sox2 and Jag1 demarcate prosensory domains and Lmx1a is relegated to non-sensory epithelium [38,44,45,46,47,48,49]. Fate mapping of Sox2 expression using a Sox2CreERT2 knock-in mouse has confirmed that the broad domain of Sox2 expressing cells present in in the early otic cup will form both future sensory and non-sensory tissues [50,51]. Over the next few days, Sox2 expression becomes restricted, and fate-mapping of these cells shows that Sox2+ progenitors contribute to less non-sensory tissue until they eventually exclusively generate sensory organs [50,51]. Similarly, timed deletions of Sox2 using the same Sox2CreERT2 results in the loss of non-sensory tissue and a lack of proper sensory organs when deleted early before the restriction of Sox2. However, deletion of Sox2 after this restriction has no discernable effect on non-sensory tissue, but now causes the loss of exclusively sensory tissue [51]. Jag1 also has a broad expression pattern throughout the otic placode and otic cup [17], but by E10.5 the expression pattern resembles that of Sox2, being largely confined to the developing prosensory domains. Like Sox2, loss of Jag1 at an early point in inner ear developments also results in the loss of both sensory and non-sensory tissue in the vestibular system, but little is known about the lineage of Jag1-expressing cells before prosensory development versus after prosensory development initiation, nor about the effects of timed Jag1 at later stages. Together this information suggests that Sox2 and Jag1 may play a role in both prosensory and non-sensory determination at the early stages, and as time passes, they become important for prosensory development. The mechanism underlying these changes is not known, but recent advances in ChIP-seq technology [52] mean that it is now possible to interrogate Sox2 targets in the otocyst at different stages in ear development to observe whether the restriction in Sox2 expression to prosensory regions is mirrored by a restriction of Sox2 binding to exclusively prosensory genes.

Jag1-mediated Notch signaling is critical for the establishment and maintenance of prosensory domains in the inner ear. Jag1 is expressed in all prosensory domains and after the domains differentiate into their respective sensory organs, it remains expressed in the supporting cells of each organ [24,53]. Jag1 acts as the main signaling ligand to Notch receptors expressed in the otocyst to create a positive feedback loop that allows for the cells of the prosensory domain to reinforce their prosensory identity as the rest of the otic epithelium proceeds down a non-sensory path [45]. This mode of Notch-mediated reinforcement of cell fate has been termed lateral induction. Notch1, Notch2, and Notch3 are all expressed in the otocyst at this stage [54], and the lateral induction mechanism, proposes that Jag1 expressed on prosensory cells binds to Notch receptors on adjacent cells and upregulates Jag1, stabilizing the prosensory identity of the cell [45]. This upregulation of Jag1 in the signal-receiving cells can then signal back to reinforce the prosensory identity of the signal sending cell, completing the loop and creating a cluster of epithelial cells in which prosensory identity is maintained.

This Jag1-mediated mode of lateral induction is supported by experiments in chick, zebrafish, and mouse. First, conditional deletion of Jag1 from an early point in otic development results in the loss or severe reduction of vestibular prosensory domain markers, the loss of vestibular sensory organs, and deformities in the vestibular architecture, with the semi-circular canals appearing to be particularly vulnerable [55,56]. Conversely, gain-of-function experiments in which Jag1 was ectopically expressed in the developing chick otocyst, result in the upregulation of Jag1 and Sox2 in adjacent cells, resulting in an ectopic prosensory domain [48,57]. When the intracellular portion of Notch1, N1ICD, is ectopically expressed in the otocyst, a similar phenomenon occurs, with Jag1 and Sox2 being upregulated both in the cells expressing N1ICD and in adjacent cells, presumably due to Jag1 signaling from the N1ICD-expressing cells [58]. Ectopic Sox2 expression also results in the production of ectopic prosensory domains, but interestingly does not upregulate Jag1, indicating that it may not be an essential component of the lateral induction machinery [59]. Together, these data indicate that lateral induction promoted by ectopic Notch signaling, can induce prosensory domain formation, and that it is required for maintaining proper prosensory domain development. It should also be noted that current conditional mutants of Jag1 do not abolish all prosensory development in the otocyst—some vestiges of vestibular sensory tissue remain, and the prosensory domain that gives rise to the organ of Corti in the cochlea appears to form, although the subsequent differentiation of the organ of Corti is highly abnormal [55]. Whether this indicates a role for additional signals in prosensory formation or is merely a consequence of the conditional mutants failing to remove all Jag1 ligand in a timely fashion is still unclear.

## 5. How Is Lateral Induction Initiated and Terminated?

Despite the clear role lateral induction plays in maintaining proper prosensory development, it is still unclear what regulates the advent of lateral induction in the otocyst, and whether a critical window exists for its action. As described above, Jag1 and Sox2 are initially expressed broadly in the otocyst, raising the question of what signals initiate their expression. Wnt signaling is known to regulate the Jag1 promoter directly [18], and manipulation of Wnt signaling can also regulate Jag1 expression in the early otocyst [17]. While it is clear lateral induction can be used to maintain prosensory domains, it would seem intuitive that lateral induction should cause the establishment of prosensory character throughout the otocyst. The solution to this paradox appears to come in the form of a non-sensory signal that opposes prosensory identity, with one of the key transcriptional effectors of this signal being Lmx1a. It has been demonstrated that Lmx1a is expressed in the non-sensory epithelium of the otocyst, with an initial stronger expression pattern on the medial side of the otocyst compared to the lateral side [26,43,49]. Loss of Lmx1a results in a deformed vestibular morphology suggestive of a failure to form semicircular canals and lack of a distinctly separated utricle and saccule, as well as enlarged vestibular sensory organs, often lacking separation between them [27,49]. Intriguingly, this expansion of sensory organs and loss of non-sensory tissue seems to be linked to Notch signaling levels in the otocyst. When a fluorescent Notch signaling reporter construct that expresses stable and unstable reporters bicistronically is introduced in the chick otocyst, some of the non-sensory epithelial cells, particularly those closer to prosensory domains, show evidence for once having received Notch signaling but subsequently ceasing reception of the Notch signal, indicating that they were initially receiving lateral inductive signals to drive them to a prosensory fate, but were then diverted to a non-sensory fate instead [49]. In addition, when Notch signaling is ectopically activated, this expands the boundaries of the prosensory domains, resulting in partially fused sensory organs like those in Lmx1a mutants [49]. Together this forms a paradigm in which Notch-mediated lateral induction in the prosensory domains competes with a non-sensory identity enforced by Lmx1a along the boundaries of the prosensory domain, setting up a specific shape for the future sensory organs. The upstream signals that induce Lmx1a expression are unclear, as is the question of whether there is a direct regulatory link between Lmx1a activity and Notch signaling genes active in lateral induction. Future efforts to identify this upstream non-sensory signal will shed light on not only the details of sensory/non-sensory fate determination but may lend insight into how and when the otocyst begins to shift to the use of Notch-mediated lateral induction.

## 6. Unresolved Questions in Notch-Mediated Lateral Induction

Although the basic parameters of Notch-mediated lateral induction have been identified, some pertinent questions remain. One of the most interesting questions is how Jag1-mediated lateral induction can operate to stabilize prosensory domains in the otocyst at the same time that Dll1-mediated lateral inhibition is acting in one of these domains—the neurosensory domain—to generate neurons. How do the cells in this domain determine which type of Notch signaling to respond to? Multiple Notch receptors are expressed in the otocyst at this time [54], but studies have also demonstrated that the intracellular domains of Notch1 and Notch2 are interchangeable and have some redundancy in the otocyst, suggesting that receptor type is unlikely to solely underlie the differential modes of Notch signaling occurring simultaneously [60,61].

During neurogenesis, cells in the otocyst that are specified as neuroblasts begin to upregulate Dll1, residing in a prosensory domain in which all cells also express Jag1. Petrovic and colleagues tested the different signaling abilities of Jag1 and Dll1 in a chick otocyst and demonstrated that Dll1 elicits a stronger Notch signal compared to Jag1, and that this stronger signaling resulted in differential activation of Notch target genes [62]. In this study, electroporation of Jag1 produces weaker Notch activity, resulting in the activation of Hey1, but only low levels of Hes5, while Dll1 produces a stronger Notch signaling activity and activates Hey1 and Hes5 in a robust fashion [62]. The ability of the otocyst to perform lateral induction and lateral inhibition was modeled in the chick, not during the neurogenic phase, but during the sensory cell differentiation phase in which the sensory cells of the inner ear, dubbed hair cells for their apical mechanosensitive stereociliary bundles, are induced to differentiate by the bHLH transcription factor Atoh1. Further modeling by this group suggested that this combination of low, prevalent Jag1 activity and high Dll1 activity, coupled with the ability of Atoh1 to induce hair cell differentiation and its own autoregulatory ability, resulted in the generation of a model that closely re-creates the pattern of hair cells surrounded by supporting cells we see in sensory organs during their development. Thus, the relative signaling ability of Jag1 plays a key role in not only prosensory domain development, but in the ability to generate the proper hair cell and supporting cell pattern of the sensory organs [62].

In addition to differential cell–cell signaling strength by different Notch ligands, post-translational modification of Notch receptors can also alter their ability to respond to Notch ligands with different affinities both between cells (*trans* interactions) and on the same cell (*cis* interactions). The Notch modifying enzyme Lunatic Fringe (Lfng) is expressed in the neurogenic/prosensory domain that forms the cochlea–vestibular ganglion and the maculae of the utricle and saccule, Lfng, is one of three known mammalian Fringe proteins, the others being Manic Fringe (Mfng) and Radical Fringe (Rfng) [63,64]. Fringe proteins act as N-acetylglucosaminyltransferases to modify Notch receptors so that they gain a stronger affinity for Delta-like ligand (Dll) signaling and a weaker affinity for Jag1 signaling in trans interactions [65,66,67], and the mechanistic basis of these interactions and how they act on different Notch receptors is beginning to be elucidated (reviewed in [63,64,68,69]). Recent work indicates that Lfng and Mfng modifications also cause Notch receptors to have a strengthened *cis*-interacting affinity for Dll1 but a weakened *cis*-interacting affinity for Jag1 [70]. These results suggest a model for neurogenic differentiation in the neurogenic–prosensory domains, and later during hair cell/supporting cell differentiation in the prosensory domain, where cells can conduct both lateral induction but also lateral induction. Most of the cells in the domain will express Notch1, Jag1, and either Lfng or Mfng, making them sensitive to strong signaling from Dll1, but experiencing minimal cis-inhibition. These cells thus maintain the ability to signal through Jag1 to achieve a low level of Notch signaling between each other and produce a distinct downstream transcriptional readout (via Hey1 and possibly other Hes/Hey genes) that supports lateral induction. Once cells in the neurosensory domain begin to express Ngn1 (or Atoh1 in the case of hair cell differentiation), those cells upregulate Dll1. Cells expressing Notch1, Dll1, and Lfng or Mfng can receive strong signals from Dll1 but not from Jag1, but can also send strong signals through Dll1 [70]. These cells that upregulate Ngn1 or Atoh1 more than their neighbors then become less responsive to the lateral inductive signals and can strongly signal to their neighbors through Dll1 and produce a downstream transcriptional readout (Hes5 and possibly other Hes/Hey genes) that supports the lateral inhibition pathway. This combination of specific ligand responsiveness and variable transcriptional response based on Notch signaling strength may help explain how lateral induction and lateral inhibition may occur simultaneously or in close temporal space (Figure 1).

Another unresolved question concerning lateral induction in the otocyst is how the very broad pattern of Sox2, Jag1, and Lmx1a expression in the early placode is downregulated and segregates into prosensory (Sox2+, Jag1+) and non-sensory regions (Lmx1a+). In a chick otic placode, Lmx1b can be positively regulated by both Fgf8 and Bmp4 [26,43], but it is not clear if these signals also regulate Lmx1a in mice, nor how the emerging cristae later express Bmp4 but repress Lmx1b. Additionally, it is unclear how much Jag1-mediated lateral induction is required to overcome the non-sensory identity induced by Lmx1a in otic epithelium outside of the sensory tissue. Understanding the changing roles of Notch signaling and Lmx1a function in the otocyst over time will require ChIP-seq experiments to determine how the targets of Lmx1a and Sox2 change with age. In addition, since the Notch transcriptional co-activator, RBPJκ, activates Notch targets in prosensory tissue in the presence of NICD but represses Notch targets in non-sensory tissue when complexed to Groucho family repressors, a comparison of RBPJκ targets in prosensory and non-sensory tissue will also be illuminating.

A final unresolved question in the role of Jag1-mediated Notch signaling in the otocyst is that the cochlear sensory epithelium appears to respond to Notch signaling differently from the other vestibular sensory regions. For example, deletion of RBPJκ or Jag1 in the otocyst does not block prosensory formation in the cochlea, although the later generation of hair cells in the organ of Corti is affected [55,71]. As the cochlea is a later evolutionary innovation in vertebrates (reviewed in [37]), it is possible that additional signals to Notch are deployed during the specification of the progenitors that will ultimately form the organ of Corti [37].

## 7. Drawing A Line in the Shifting Sands: Notch Signaling Plays a Role in Boundary Formation in the Developing Cochlea

As mentioned above, the cochlea shares some of its developmental characteristics with the vestibular sensory domains, but there are some significant differences in not only gene expression pattern, but the employment of those developmental processes. This is not an entirely unexpected scenario when we consider the evolution of the basilar papilla (the tetrapod hearing organ) in tetrapods, which is then even further modified in mammals as the organ of Corti, with current evidence linking the rise of the basilar papilla to an outgrowth and widening of a ventral sensory papilla in the common tetrapod ancestor [72,73,74]. From there, we know that the mammalian cochlea appears to be distinctly different from other vertebrate hearing structures, as even in the basal egg-laying mammals it has a highly organized pattern of inner hair cells and outer hair cells surrounded by specialized supporting cells, and in true mammals is accompanied by a characteristic coiling and loss of the lagenar macula apically [72]. While birds do possess a gradient of “tall” hair cells to “short” hair cells, this gradient is smooth, as compared to the stark divide between inner and outer hair cells; and even though avian hair cells express some of the genes important for outer hair cell function like prestin, there does not appear to be a difference in the expression of these genes in the basilar papilla between tall and short hair cells [75]. Comparative vertebrate studies suggest that the mammalian cochlea is unique in form to that clade, and the way the cochlear sensory organ develops compared to the rest of the inner ear lends this some credence [76].

Many genes important for vestibular prosensory development are redeployed in the cochlea during development, albeit with a different expression pattern. BMP4, FGF10, Lfng, Sox2, and Jag1 are all expressed in the developing cochlea, though most have a dynamic expression pattern as a ribbon of cells running along the length of the cochlear duct is specified to form a prosensory domain that will become the organ of Corti (reviewed in [20]). Unlike other prosensory domains where hair cells and supporting cell progenitors exit the cell cycle shortly before differentiating, cells of the cochlear prosensory domain exit the cell cycle in an apical to basal manner [77,78], but then differentiate into the hair and supporting cells in a basal to apical manner [79,80]. For simplicity, the following expression pattern descriptions refer to the basal/mid-basal region of the cochlea with regard to timing, with events occurring with a slight delay as one moves progressively more apically.

When the developing cochlear duct is viewed in cross-section, at E11.5 the expression of Sox2, Jag1, and BMP4 is spread throughout the epithelium that will eventually form Kolliker’s organ, the organ of Corti, and the outer sulcus (referred to in order from the neural side, where spiral ganglion neurons invade and innervate hair cells, to the abneural side) [81]. FGF10 and Lfng are expressed more strongly on the neural side than the abneural side, with FGF10 having the more constricted expression pattern of the two [14,81]. As the cochlea begins to grow out further from the otocyst and coil, the expression pattern of each of these genes begins to constrict. By E13.5, the expression of Jag1 and Sox2 are no longer expressed in the most abneural region, with Sox2 downregulated from the neural-most side and Jag1 only having a small overlapping expression pattern with Sox2, but otherwise being excluded from the centrally located Sox2-expressing domain. While FGF10 seems to maintain the same expression pattern from the neural-most epithelium to roughly the center (overlapping with Jag1), Lfng has begun to constrict slightly from the neural-most side leaving a small domain at the neural-most bit of epithelium and otherwise expressing in a region most closely resembling the region of overlap between Sox2 and Jag1 [82]. At this time the prosensory domain is established: Cells have exited the cell cycle and express Sox2, with a small portion of the prosensory domain on the neural side expressing Jag1 and Lfng. These features set up the cochlear prosensory domain so that it may develop asymmetrically, and allow Notch signaling to establish a boundary where the inner hair cells of the organ of Corti begin to differentiate.

By the time the cochlear prosensory domain has exited the cell cycle at E14.5, the cochlear duct can be divided into three broad regions, the greater epithelial ridge (GER), the now-postmitotic prosensory domain that will become the organ of Corti, and the outer sulcus [81]. The organ of Corti consists of one row of inner hair cells surrounded by their associated supporting cells, the inner phalangeal cells. A second group of supporting cells, pillar cells, create a boundary between inner hair cell and outer hair cell compartments. Finally, the outer hair cells are surrounded by their own supporting cells, Deiters’ cells, and the remaining supporting cells on the abneural side of the outer hair cells are called Hensen’s cells and Claudius’ cells. Notably, the first hair cells to develop in the cochlea are the inner hair cells, which form at the border of the prosensory domain where Sox2 and Jag1 overlap [82]. Hair cell and supporting cell differentiation occurs in inner ear prosensory domains through a second deployment of Notch-mediated lateral inhibition. The basic principles of lateral inhibition that govern neurogenesis described above apply to hair cell and supporting cell differentiation with some key differences. Instead of Ngn1, the bHLH transcription factor Atoh1 is the primary hair cell fate determining gene, and it positively regulates the expression of the Notch ligands Jagged2 (Jag2) and Dll1, while being negatively regulated by Notch signaling itself [83,84,85]. The loss of Atoh1 results in prosensory domains with no hair cells, while ectopic Atoh1 expression is in some cases sufficient for the formation of hair cell-like cells [86,87]. Prosensory domain cells in the mouse otocyst begin to upregulate Atoh1 around E11.5 in the vestibular system and around E13.5 in the cochlear duct [23,79,88]. As one would expect, the loss of prosensory character via the early loss of Sox2 results in inner ears devoid of hair cells [51,89]. When Notch signaling is disturbed in this system, developing hair cells can no longer inhibit their neighbors from becoming hair cells fate resulting in the generation of extra hair cells at the expense of supporting cells. In the cochlea, progressive deletion of Jag2 and Dll1 alleles leads to the formation of increasing numbers of supernumerary inner and outer hair cells, while deletion of Notch1 (either genetically or through chemical or immunoreactive inhibition) causes significant numbers of supernumerary hair cells, suggesting that Notch1 is the principal receptor for Dll1 and Jag2 in the prosensory domain [61,90].

Although Dll1 and Jag2 act to specify the fine-grained pattern of hair cells and supporting cells, recent evidence from Basch and colleagues suggest that Fringe proteins and Jagged1 play a role in establishing the boundary for hair cell differentiation between the GER and the prosensory domain [82]. Detailed expression analysis show that Lfng and Mfng expression coincide at a very narrow band on the neural edge of the cochlear prosensory domain just as hair cell differentiation commences, resulting in a band of cells that express Lfng, Mfng, Sox2, Jag1, and Atoh1 bordered by cells expressing Jag1 and Sox2 on the GER side and prosensory side [82]. The loss of both Lfng and Mfng in combination results in the duplication of inner hair cells and their associated inner phalangeal cells, similar to that seen in Jagged mutants and low levels of pharmacological Notch signaling inhibition via the Notch signaling inhibitor DAPT [55,82,90]. This suggests a model in which cells at the prosensory domain border with the GER can strongly inhibit the cells in the GER, but cells in the GER are not able to strongly signal back to the prosensory border cells (as Lfng and Mfng-modified Notch receptors have a strong affinity for Dll1 but not Jag1). These border cells can thus force GER cells away from a sensory fate, and still signal between one another via Dll1 to establish hair cell/supporting cell fate decisions. These first hair cells to differentiate will become the inner hair cells, and when Notch signaling is reduced by reducing ligands or the responsiveness of the Notch receptors during this phase, it results in the ability of GER cells along the prosensory border to also become hair cells, resulting in an inner hair cell duplication phenotype. In this way, small disturbances in Notch signaling result in disruptions of the number of inner hair cells specifically at this border, while more severe disturbances in Notch signaling result in supernumerary inner and outer hair cells and an almost complete loss of supporting cells.

## 8. A Future for Notch? Notch Signaling In the Inner Ear after Sensory Cell Development

Once Notch-mediated lateral inhibition specifies the correct proportion of hair cells and supporting cells in the cochlea, the organ of Corti still continues to mature, but no more hair cells or supporting cells are generated. Despite this, Notch signaling receptors and ligands are present to varying degrees during this maturation time, particularly Jag1, which localizes to supporting cells after differentiation in the organ of Corti along with Lfng, while Notch1, Dll1, Jag2, and Mfng localize to the hair cells [53,54,82,91]. Understanding the function of Notch signaling after hair cell differentiation is relevant to efforts to regenerate hair cells after they have been damaged or lost. The mammalian inner ear differs from the inner ears of other vertebrate lineage in that it appears to have a very limited ability to regenerate its hair cell populations after they have been killed. In addition to hair cell loss through the normal process of aging, hair cells can also be killed by sustained high occupational or recreational noise, and by ototoxic antibiotics and chemotherapy drugs. Non-mammalian vertebrates such as birds and fish can regenerate their hairs cells through one of two processes: 1) use of supporting cells as a stem cell niche, in which supporting cells divide asymmetrically to give a hair cell and a supporting cell; or 2) direct transdifferentiation, in which supporting cells differentiate directly into hair cells without division. It has been observed after the loss of hair cells occurs in birds that the supporting cells begin to upregulate Atoh1 and Dll1, two of the key genes needed for Notch-mediated lateral inhibition to occur and set up the fate decisions between hair cells and supporting cells, implicating the re-use of the original developmental programming used to generate the sensory organ of the basilar papilla [62]. Mammals are unable to naturally regenerate the hair cells of the cochlea in any functionally significant manner, although there is evidence that the vestibular sensory organs possess a limited ability to regenerate some hair cells after genetic ablation with diphtheria toxin [92]. Determining the mechanistic basis for these differences, and what role Notch signaling may play in this process, will provide valuable information for efforts to promote hair cell regeneration in mammals [93,94].

The potential role of Notch signaling in hair cell development and regeneration has been examined both postnatally and in the mature cochlea. It has been demonstrated that Notch signaling has different effects on the development of hair cells, depending on their developmental age. Thus, when N1ICD is expressed as hair cells begin to differentiate, hair cells begin to upregulate supporting cell-specific genes and the animals become functionally deaf with the hair cells dying, but if N1ICD is expressed in hair cells after they differentiate, they become largely unresponsive to Notch signaling-induced fate changes, upregulating the supporting cell genes Sox2 and Prox1, but otherwise showing no other detectable effects on hair cell maturation [95,96]. Over-expression of N1ICD in supporting cells of neonatal mice also reduces the spontaneous regeneration of hair cells that can occur in early postnatal life [97]. Loss of Notch1, Dll1, Jag2, or other Notch pathway genes during hair cell differentiation results in the generation of supernumerary hair cells (see Table 1), as does the inhibition of gamma secretases through pharmacological agents like DAPT [61,90]. However, after birth, blocking Notch signaling by deletion or inhibition of receptors and ligands has less and less effect on the pattern of hair cells and supporting cells. Notch1, Notch3, Jag1, Jag2, Dll1, and multiple Hes/Hey genes are known to be expressed in the organ of Corti through at least P6, although their levels decline after birth [90]. The use of Notch1 blocking antibodies demonstrated that Notch1 is the significant receptor during postnatal Notch signaling activity, as Notch1 blockage leads to the number of supernumerary hair cells seen with gamma secretase inhibitors [90]. Additional efforts to manipulate Notch signaling to promote hair cell regeneration have made use of Wnt activation to enhance regeneration in neonatal mice [93,98]. A number of studies have combined activation of Wnt signaling and inhibition of Notch signaling, either genetically or pharmacologically, to demonstrate the proliferation of supporting cells and their trans-differentiation to hair cells in the neonatal cochlea [99,100,101].

As the organ of Corti matures into adulthood, the levels of Notch pathway genes continue to drop, although there is some indication that Jag1 remains expressed at decreased but observable levels in supporting cells, together with the proposed “low-strength” Notch signaling target Hey1 [53,90]. This suggests the possibility of some form of low-strength Notch signaling activity in the adult organ of Corti, but efforts to interrogate its presence and role remain inconclusive. Consequently, efforts to manipulate Notch signaling to create new hair cells in the adult animal have given mixed and conflicting results. Inhibition of Notch signaling with gamma secretase inhibitors after noise damage to kill hair cells in mice has been reported to generate small numbers of new hair cells in the cochlea, suggesting that some level of Notch signaling inhibits supporting cells from becoming hair cells in the adult organ of Corti [83]. However, other evidence strongly suggests that not only are key Notch signaling genes expressed at notably lower levels in the adult cochlea, but that the ability of gamma secretase inhibition to generate supernumerary hair cells declines precipitously by P6 [90]. The use of general gamma secretase inhibitors is also complicated by the fact that gamma secretases cleave scores of cell surface proteins in addition to Notch receptors [102]. Interestingly, a recent study demonstrated that activation of the Notch pathway through induction of N1ICD coupled with the transduction of the Myc transcriptional regulator led to the proliferation of adult cochlear supporting cells, and transduction of these with the Atoh1 transcription factor generated new hair cells [103]. Nevertheless, the consensus view of these studies suggest that as time passes and the organ of Corti matures, the role of Notch signaling changes over time. Viewed in this context, Notch signaling may act in a manner analogous to a construction scaffold—it specifies hair cell and supporting cell fates during development, but is not required to maintain these fates in the adult and is therefore dismantled by downregulation. This could be explained by changes in the epigenetic state of hair cell and supporting cell genes as the organ of Corti matures [104]. This raises the question of whether modifying the chromatin structure to be more accessible at hair cell gene loci and then modifying Notch signaling activity in the mature cochlea may lead to hair cell regeneration.

**Table 1:** Summary of mouse Notch pathway mutants that affect inner ear development.

Notch Receptors and Ligands	Type of Mutation	Phenotype	Reference
Notch1	Inner ear-specific knockout with Foxg1-Cre or Pax2-Cre	Many cochlear supporting cells (with the exception of inner pillar cells) convert to ectopic inner and outer hair cells	[61,82]
Jag1	Inner ear-specific knockout with Foxg1-Cre	Severe loss of semicircular canals and small or absent vestibular sensory organs. Cochlea has either reduced or absent outer hair cells and supernumerary inner hair cells.	[55,56]
Jag1	*Headturner* allele; ENU-induced mutation (G289D)	Truncated anterior and/or posterior semicircular canals, loss of some outer hair cells, supernumerary inner hair cells.	[91]
Jag1	*Ozzy* allele; ENU-induced mutation (W167R)	Variably truncated semicircular canals	[105]
Jag1	*Slalom* allele; ENU-induced mutation (P269S)	Truncated anterior and/or posterior semicircular canals, loss of some outer hair cells, supernumerary inner hair cells.	[91]
Jag1	*Nodder* allele; ENU-induced mutation (H268Q)	Vestibular defects (head nodding)	[106]
Jag2	Null mutant	Supernumerary inner and outer hair cells and inner phalangeal cells.	[82,107]
Dll1	Inner ear-specific knockout with Foxg1-Cre	Supernumerary inner and outer hair cells and a small increase in supporting cells	[55]
Dll3	Null mutant	Despite expression in hair cells, no hair cell phenotype	[108]
**Notch Transcriptional Co-Activators**	**Type of Mutation**	**Phenotype**	**Reference**
RBPJk	Inner ear-specific knockout with Foxg1-Cre or Pax2-Cre	Severe loss of semicircular canals and small or absent vestibular sensory organs. Cochlea shows evidence of supernumerary inner hair cells but mice die before this becomes patent	[71,109]
MAML1-3	Activation of dnMAML allele with Pax2-Cre	Supernumerary inner hair cells and inner phalangeal cells.	[79]
**Notch Modifying Enzymes**	**Type of Mutation**	**Phenotype**	**Reference**
Pofut1	Inner ear-specific knockout with Pax2-Cre	Supernumerary inner and outer hair cells and inner phalangeal cells.	[79]
Lfng	Null mutant	Single mutants have no cochlear phenotype; double mutants have supernumerary inner hair cells and inner phalangeal cells.	[79]
Mfng	Null mutant		
Lfng; Mfng	Null mutant		
Lfng; Jag2	Null mutants	The Lfng mutant allele rescues the Jag2 mutant phenotype in the inner hair cell region but not the outer hair cell region	[110]
**Notch Downstream Targets**	**Type of Mutation**	**Phenotype**	**Reference**
Hes1	Null mutant	Increasing severity of supernumerary inner and outer hair cells with increasing combinations of multiple mutant alleles; Hes1;Hes5;Hey1 triple mutants having the most severe phenotype [102]	[87,111,112,113,114,115]
Hes5	Null mutant		
Hey1	Null mutant		
HeyL	Null mutant		
Hey2	Null mutant	No significant phenotype in null; however pharmacological inhibition of Notch signaling in Hey2 mutants causes inner pillar cells to convert to hair cells.	[114]

## 9. Conclusions

The inner ear, like many organs in the animal kingdom, uses Notch signaling at multiple points in development. Notch signaling serves to first establish otic identity, generate the neurons that will later innervate otic sensory organs, help form and maintain the prosensory domains that will become the sensory organs, set up a boundary of differentiation in the cochlea, serve as a means of determining cell fate during hair cell and supporting cell differentiation, and help maintain the relationship between hair cells and supporting cells as the inner ear’s sensory organs mature. Notch signaling is employed in at least two different modes of action, lateral induction and lateral inhibition, during this developmental time course, sometimes simultaneously. The stereotypical and crystalline arrangement of cell types in the inner ear offers an attractive model for exploring questions about the nature of these modes of Notch signaling and how they interact with one another.
